# CalliSpheres drug-eluting beads versus lipiodol transarterial chemoembolization in the treatment of hepatocellular carcinoma: a short-term efficacy and safety study

**DOI:** 10.1186/s12957-018-1368-8

**Published:** 2018-03-27

**Authors:** Baolin Wu, Jun Zhou, Gonghao Ling, Dongyong Zhu, Qingyun Long

**Affiliations:** Department of Radiology, Zhongnan Hospital of Wuhan University, No. 169 Donghu Road, Wuhan, 430071 Hubei People’s Republic of China

**Keywords:** Hepatocellular carcinoma, Transarterial chemoembolization, Efficacy, Safety, Drug-eluting beads, CalliSpheres Beads

## Abstract

**Background:**

The present study aimed to evaluate the short-term efficacy and safety of drug-eluting beads transarterial chemoembolization (DEB-TACE) with CalliSpheres Beads loaded with doxorubicin (DOX) in the treatment of Chinese patients with hepatocellular carcinoma (HCC) compared to conventional TACE (cTACE).

**Methods:**

A total of 54 patients with HCC treated by TACE from June 2016 to February 2017 were retrospectively analyzed. These included 24 cases in the DEB-TACE group and 30 cases in the cTACE group. The clinical efficacy, tumor recurrence rate, and complications were compared between the two groups. Furthermore, liver function tests and alpha-feto protein levels were compared between the two groups before and at 1 week and 1 month after interventional treatment.

**Results:**

There were no significant differences in baseline characteristics (*p* > 0.05). Tumor response rates and disease control rates in the DEB-TACE group were significantly higher than those in the cTACE group at 3 and 6 months after treatment (*p* < 0.05). Recurrence rates at 6 months were significantly higher for cTACE compared to DEB-TACE (43.3 vs. 16.7%; *p* = 0.036). At 1 month, the AFP level in the DEB-TACE group was significantly lower than that in the cTACE group (*p* = 0.008). At the end of follow-up, four cases in the DEB-TACE group and two cases in the cTACE group were treated with salvage surgery after downstaging the disease. Liver function of both groups improved at 1 month. However, alanine aminotransferase, aspartate aminotransferase, and total bilirubin levels in the DEB-TACE group were better than those in the cTACE group (*p* < 0.05). The incidence of DOX-related complications in the DEB-TACE group was significantly lower than in the cTACE group (*p* < 0.05).

**Conclusion:**

The short-term efficacy of DEB-TACE is better, and the complication rates are lower compared to cTACE in the treatment of Chinese patients with HCC. However, long-term clinical efficacy and survival benefit should be analyzed in future studies.

## Background

The incidence of hepatocellular carcinoma (HCC) has continued to rise over the past decades due to increasing numbers of hepatitis B (HBV) and C virus (HCV) infections [1]. HCC has become one of the major causes of cancer-related deaths worldwide [2]. Curative treatments for early-stage HCC include surgical treatment (liver resection and liver transplantation) and local therapy (radiofrequency ablation). However, when diagnosed with HCC, approximately 70% of cases are at later stages according to the Barcelona Clinic Liver Cancer (BCLC) staging system, and it is not suitable for these patients to be treated with surgical resection [3, 4]. Liver transplantation is an effective method for the treatment of HCC, but donors for liver transplantation are scarce and the cost is very high, which limits its use for the treatment of HCC. Currently, transarterial chemoembolization (TACE) has been generally considered as an effective treatment option for unresectable HCC [5]. As a locoregional therapy, TACE plays an important role in the management of patients at non-early stages. It has been used not only as a palliative treatment for advanced cases but also as a downstaging/bridging/necrotizing tool prior to a further surgical resection or liver transplantation. However, conventional TACE (cTACE) often uses emulsions of lipiodol with doxorubicin (DOX) or other chemotherapy drugs, which do not reside long in tumor tissues and will rapidly enter the systemic blood circulation. Thus, cTACE will reduce the local antitumor drugs concentration, but at the same time, easily cause systemic adverse events.

Drug-eluting beads transarterial chemoembolization (DEB-TACE) has been used as a novel drug delivery and embolization system in recent years, which can not only load chemotherapy drugs and release them slowly in local regions but can also embolize the tumor supply vessels permanently [6–8]. Clinical trials showed that DOX-loaded DEB-TACE resulted in higher intra-tumoral drug concentrations and lower systemic toxicity [9, 10].

At present, DEB products have not been widely used in China. CalliSpheres Beads (CB) is the first DEB product in China and has just been applied to clinical use in the last 2 years. Studies associated with CB are limited. Therefore, the clinical efficacy and safety of CB in Chinese patients with HCC are still unclear.

The aim of this study is to compare the short-term clinical efficacy and safety of DEB-TACE with CB loaded with DOX and cTACE in the treatment of HCC to analyze CB’s clinical value and safety in Chinese patients with HCC.

## Methods

### Patients and groups

The institutional review board of the Zhongnan Hospital of Wuhan University approved this retrospective study and waived the requirement for patient informed consent. A total of 84 patients diagnosed with unresectable HCC by imaging, serological, and pathological examinations were treated in our hospital from June 2016 to February 2017. The inclusion criteria were (1) primary interventional treatment, (2) aged 18 to 75 years, (3) BCLC stage B or C, (4) Child-Pugh grade A or B, (5) Eastern Cooperative Oncology Group performance status (ECOG PS) 0–2, (6) patients without portal vein, vena cava or bile duct thrombi, and (7) the expected survival time was more than 6 months. The exclusion criteria were (1) malignant tumor of other sites, (2) other treatment or antineoplastic drugs were performed for the corresponding period, and (3) unstable systemic disease or uncontrolled infection.

### TACE procedures

After local disinfection and anesthesia, percutaneous right femoral artery puncture intubation with a modified Seldinger technique was performed, and the left femoral artery or the left brachial artery was punctured, if necessary. Then, a 5F-Yashiro or RH (Terumo, Japan) catheter was introduced through a 5-F vascular sheath and placed into the common hepatic artery under DSA guidance to perform celiac angiography to identify the hepatic arterial anatomy and the potential existence of variants. Tumor locations, sizes, and numbers, as well as portal vein tumor thrombus and hepatic arteriovenous fistula, were identified through initial angiography. If there was no definite tumor-feeding artery, angiography of the superior mesenteric artery, bilateral inferior phrenic artery, internal thoracic artery, and adrenal artery should be performed to confirm the tumor-feeding artery. Subsequently, a 2.7-F Progreat microcatheter (Terumo, Japan) was advanced super-selectively into the right or left hepatic artery or segmental tumor-feeding artery through a coaxial hydrophilic guidewire.

1. cTACE group: after fully mixed by the pumping method, emulsions of lipiodol (5–15 ml) and DOX (10–20 mg) were slowly injected into the tumor-feeding artery through a microcatheter under fluoroscopic monitoring to avoid reflux of lipiodol emulsion followed by the infusion of a gelatin sponge. The TACE procedure was terminated when target blood flow interruption or tumor stain disappearance was observed. The dosage of chemotherapeutic drugs was adjusted according to the patient’s liver function tests. The amount of lipiodol and chemotherapeutic drug emulsions were given according to the tumor size and tumor-feeding arterial blood flow.
2. DEB-TACE group: CalliSpheres Beads (Jiangsu Hengrui Medicine Co. Ltd., Jiangsu, China) were used as the carrier to load DOX with sizes of 300–500 μm or 100–300 μm, depending on the tumor sizes and blood supply. 30 min were required to load DOX with a dosage of 60–80 mg every 20 ml CB prior to the beginning of the TACE procedure. During the loading procedure, the mixture was shaken every 5 min at room temperature (20–25 °C) to enable the CB to fully load with DOX. Once loaded, iodine alcohol, a non-ionic contrast agent, was mixed with CBDOX (at a volume ratio of 1:1). Then, the CBDOX was manually injected into the tumor-feeding artery slowly and carefully under fluoroscopic monitoring to avoid reflux of the CBDOX into non-target vessels. Subsequent angiography was performed to identify the extent of vascular occlusion.

### Evaluation of clinical efficacy

Individualized computed tomography (CT) or magnetic resonance imaging (MRI) was performed within 1 week prior to initial TACE to evaluate the baseline tumor imaging. Follow-up individualized CT or MR was performed at 3 and 6 months after treatment to assess the local tumor response according to the modified Response Evaluation Criteria in Solid Tumors (mRECIST [11]). Objective response (OR) was defined as complete remission (CR) plus partial remission (PR), and disease control (DC) was defined as CR, PR plus stable disease (SD). Progression of disease (PD) was defined as local recurrence and new lesions, as well as a combination of both (overall recurrence). The intrahepatic tumor lesions were evaluated by two experienced (more than 5 years working experience) abdominal radiologists in cooperation with our department. TACE was repeated in cases with local tumor progression and without contraindications. Tumor recurrences were recorded at each follow-up time point. Alpha-fetoprotein (AFP) levels were also recorded before and at 1 week and 1 month after interventional treatment.

### Safety evaluation

Safety was evaluated by monitoring changes in liver enzymes at 1 week and 1 month after treatment. Parameters related to liver function included serum albumin (ALB), total protein (TP), alanine aminotransferase (ALT), aspartate aminotransferase (AST), and total bilirubin (TBiL) levels. Complications associated with the TACE treatment were recorded during the follow-up period and included liver toxicity, post-embolization syndrome (including nausea, vomiting, fever, and abdominal pain), liver abscess, ascites, and DOX-related complications.

### Statistical analysis

SPSS Statistics package (version 22.0; IBM, Chicago, IL) was used for statistical analyses. All data of continuous variables were expressed as the mean ± standard deviation. Numerical differences between groups were assessed by chi-square test for categorical variables and *t* test for continuous variables. Statistical significance was set at *p* < 0.05.

## Results

### Baseline clinical characteristics

A total of 54 patients with unresectable HCC were eventually enrolled in this retrospective study, including 24 treated by DEB-TACE (DEB-TACE group; 22 males and 2 females; mean age ± standard deviation, 56.25 ± 7.47; age range, 43–75 years; mean tumor diameter ± SD, 7.25 ± 2.33 cm) and 30 treated by cTACE (cTACE group; 27 males and 3 females, mean age ± standard deviation, 52.8 ± 6.13; age range, 46–74 years; mean tumor diameter ± SD, 7.53 ± 2.39 cm). For 46 of these patients, TACE was the first treatment, and eight were treated after postoperative recurrence. There were no significant differences in baseline clinical and demographic characteristics between the two groups (Table 1). There were no deaths, and no patients were lost in the 6 months follow-up.

**Table 1:** Comparison of baseline clinical characteristics of patients between DEB-TACE and cTACE

Variables	DEB-TACE	cTACE	*χ*^*2*^ or *t* value	*p* value
	*n* = 24	*n* = 30		
Gender (M/F)	22/2	27/3	0.044	0.834^*^
Age (mean ± SD)	56.25 ± 7.47	52.83 ± 6.13	0.225	0.823^#^
Child-Pugh				
A	10 (42%)	14 (47%)	0.135	0.713^*^
B	14 (58%)	16 (53%)		
BCLC stage				
B	13 (54%)	17 (57%)	0.034	0.854^*^
C	11 (46%)	13 (43%)		
ECOG PS				
0 ~ 1	15 (63%)	18 (60%)	0.035	0.851^*^
2	9 (37%)	12 (40%)		
Etiology				
HBV	15 (62.5%)	18 (60.0%)	0.035	0.851^*^
HCV	5 (20.8%)	4 (13.3%)	0.540	0.462^*^
Alcohol	1 (4.2%)	2 (6.7%)	0.159	0.690^*^
Other and mixed	3 (12.5%)	6 (20.0%)	0.540	0.462^*^
Amount of tumors				
1~2	16 (66.7%)	19 (63.3%)	0.065	0.799^*^
3	8 (33.3%)	11 (36.7%)		
Tumor diameter mean ± SD (cm)	7.25 ± 2.33	7.53 ± 2.39	−0.438	0.663^#^

*χ^*2*^ test, ^*#*^*t* test

BCLC Barcelona clinic liver cancer, ECOG PS eastern cooperative oncology group performance status, HBV hepatitis B virus, HCV hepatitis C virus

### Curative effect

**Fig. 1:**
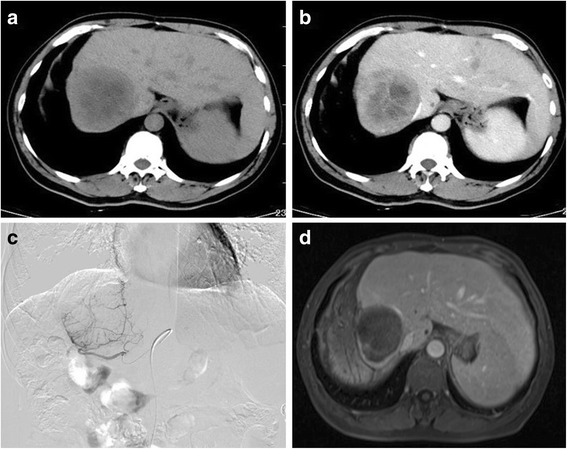
Phases of DEB-TACE with CBDOX in a 54-year-old male patient. **a–b** Plain phase and portal phase of pretreatment CT scan revealed a recurrence lesion located at the right lobe of the liver 11 months after surgical resection. **c** Angiography during the DEB-TACE procedure revealed a comparatively hypervascular lesion. **d** An enhanced MRI scan performed at 3 months after treatment showed a complete remission to treatment

The local tumor response rates are summarized in Table 2. CR (Fig. 1), PR, and SD in the DEB-TACE group were achieved in 6, 14, and 2 patients at 3 months and 5, 10, and 5 at 6 months, respectively, which reached OR and DC rates of 83 and 92% at 3 months and 63 and 83% at 6 months, respectively. For the cTACE group, CR, PR, and SD were achieved in 1, 12, and 7 patients at 3 months; no patients achieved CR; 9 patients achieved PR; and 8 patients achieved SD at 6 months, with OR and DC rates of 43 and 67% at 3 months and 30 and 57% at 6 months, respectively. CR, OR, and DC rates in the DEB-TACE group were all significantly higher than those in cTACE group at 3 and 6 months. Furthermore, PD rate at 3 months (*p* = 0.028) and 6 months (*p* = 0.036) were significantly lower in the DEB-TACE group than in the cTACE group. Overall, local response achieved at 3 and 6 months follow-up were better in the DEB-TACE group compared to the cTACE group.

**Table 2:** Local tumor response at 3 months and 6 months after treatment

Treatment	3 months						6 months					
	CR	PR	SD	PD	OR	DC	CR	PR	SD	PD	OR	DC
DEB-TACE (*n* = 24)	6 (25.0%)	14 (58.3%)	2 (8.3%)	2 (8.3%)	20 (83.3%)	22 (91.7%)	5 (20.8%)	10 (41.7%)	5 (20.8%)	4 (16.7%)	15 (62.5%)	20 (83.3%)
cTACE (n = 30)	1 (3.3%)	12 (40.0%)	7 (23.3%)	10 (33.3%)	13 (43.3%)	20 (66.7%)	0	9 (30.0%)	8 (26.7%)	13 (43.3%)	9 (30.0%)	17 (56.7%)
*χ*^*2*^ value	5.548	1.795	2.160	4.821	8.977	4.821	6.888	0.796	0.248	4.396	5.704	4.396
*p* value	0.019	0.180	0.142	0.028	0.003	0.028	0.009	0.372	0.618	0.036	0.017	0.036

CR complete response, PR partial response, SD stable disease, PD progressive disease, OR objective response, DC disease control

Overall tumor recurrence occurred in 8.3% at 3 months and reached 16.7% at 6 months in the DEB-TACE group. In the cTACE group, the respective tumor recurrence rates were 33.3 and 43.3%. The overall tumor recurrence rates at 3 and 6 months in the DEB-TACE group were both significantly lower compared to the cTACE group (*p* = 0.028 and *p* = 0.036, respectively) (Table 3).

**Table 3:** Comparison of tumor recurrence rates of HCC between DEB-TACE and cTACE

Group	Tumor recurrence	3 months	6 months
DEB-TACE (*n* = 24)	Local recurrence	1	4
	New lesions	1	2
	Overall rate^a^	8.3% (2/24)	16.7% (4/24)
cTACE (*n* = 30)	Local recurrence	9	13
	New lesions	5	7
	Overall rate^a^	33.3% (10/13)	43.3% (13/30)
	Local recurrence	0.015	0.036
*p* value	New lesions	0.146	0.142
	Overall rate^a^	0.028	0.036

^a^Local recurrence and new lesions may coexist, it means the number of patients, not the total of recurrences

AFP levels during the study are shown in Table 4. The mean values of the AFP levels were 1197.41 ± 628.81 ng/ml, 566.22 ± 217.99 ng/ml, and 158.06 ± 69.89 ng/ml in the DEB-TACE group and 1144.69 ± 444.35 ng/ml, 604.91 ± 250.00 ng/ml, and 221.00 ± 109.12 ng/ml in the cTACE group at baseline, 1 week and 1 month, respectively. There was a statistically significant decrease in the levels of AFP after 1 week and 1 month of treatment in both groups. At 1 month, the AFP level in the DEB-TACE group was significantly lower than that in the cTACE group (*p* = 0.008), indicating a relatively better response of the tumors to the CBDOX.

**Table 4:** Liver enzymes and AFP levels changes before and after treatment

Variables	DEB-TACE (*n* = 24)			cTACE (*n* = 30)			DEB-TACE vs cTACE^c^	
	Before TACE	1 week after TACE^a^	1 month after TACE^b^	Before TACE	1 week after TACE^a^	1 month after TACE^b^	*t* value	*p* value
ALB (g/L)	40.64 ± 4.77	34.07 ± 4.42	38.23 ± 3.83	39.64 ± 4.33	34.45 ± 3.74	37.52 ± 4.05	0.654	0.516
TP (g/L)	66.32 ± 7.32	61.06 ± 5.27	65.55 ± 5.62	67.03 ± 4.23	59.11 ± 4.35	64.31 ± 3.05	1.037	0.304
ALT (U/L)	38.17 ± 22.92	58.38 ± 38.08	40.50 ± 22.23	38.04 ± 15.71	65.92 ± 30.83	56.75 ± 30.80	− 2.057	0.045
AST (U/L)	44.58 ± 25.06	63.00 ± 25.52	45.67 ± 15.51	45.47 ± 32.53	73.87 ± 47.64	60.77 ± 32.30	− 2.102	0.040
TBil (umol/L)	16.11 ± 4.28	30.33 ± 18.06	16.85 ± 4.24	16.29 ± 4.63	29.59 ± 16.61	20.87 ± 8.47	− 2.124	0.038
AFP (ng/ml)	1197.41 ± 628.81	566.22 ± 217.99	158.06 ± 69.89	1144.69 ± 444.35	604.91 ± 250.00	221.00 ± 109.12	− 2.748	0.008

^a^Compared with before treatment, *p*<0.05

^b^Compared with 1 week after treatment, *p*<0.05

^c^Comparison between DEB-TACE and cTACE at 1 month by using *t* test

At the end of follow-up, a total of 6 patients, including 4 cases (16.7%, 4/24) in the DEB-TACE group and 2 cases (6.7%, 2/30) in the cTACE group were also treated with salvage surgery after downstaging the disease. The remaining cases that were not suitable for hepatic resection (HR) received TACE only. Hepatic resections were all successfully performed, and the mean time interval between TACE and HR was 2.9 ± 1.8 months. At the end of follow-up, no patients received a liver transplant.

### Safety

There were no significant differences in liver function tests between the two groups before treatment (*p* > 0.05). At 1 week after intervention, ALB and TP levels were significantly decreased in the two groups, while ALT, AST, and TBiL levels were significantly increased. At 1 month after treatment, liver function tests improved in the two groups. However, ALT (*p* = 0.045), AST (*p* = 0.040), and TBiL (*p* = 0.038) levels in the DEB-TACE group were significantly lower than those in cTACE group. There were no significant differences in ALB and TP levels between the two groups (*p* > 0.05) (Table 4).

The most common complication in the two groups was post-embolization syndrome, which occurred in 15 patients in the DEB-TACE group and 26 patients in the cTACE group; this was a significant difference (*p* = 0.015) (Table 5). The incidences of DOX-related complications, including bone marrow suppression (*p* = 0.015) and granulocyte reduction (*p* = 0.004), in the DEB-TACE group were significantly lower than those in the cTACE group (Table 5). There were no significant differences in the incidence of other complications (*p* > 0.05)*.*

**Table 5:** Comparison of complications induced by TACE therapy between DEB-TACE and cTACE

Complications	DEB-TACE	cTACE	*χ*^*2*^ value	*p* value
Post-embolization syndrome	15 (62.5%)	26 (81.3%)	4.260	0.039
Transient liver injury	11 (46%)	20 (67%)	2.367	0.124
Liver abscess	1 (4.1%)	0	1.274	0.259
Ascites	3 (13%)	8 (27%)	1.650	0.199
Myelosuppression	1 (4.1%)	9 (30%)	5.897	0.015
Granulocyte reduction	2 (8.3%)	13 (43%)	8.142	0.004

## Discussion

Traditionally, cTACE is usually performed by using lipiodol mixed with chemotherapeutic agents to block the tumor-feeding arteries and increase the concentration of chemotherapeutic agents in tumor tissue, while the surrounding normal liver parenchyma areas are protected from chemotherapy toxicity. With this strategy, lipiodol is considered not only an embolic agent but also a carrier of chemotherapeutic agents [12, 13]. A previous study has suggested the conferred survival benefit of cTACE treatment in HCC compared to conservative treatment [14]. However, the tumor recurrence rate was relatively high after cTACE [15–17].

The optimal embolic material should achieve a higher concentration of chemotherapy agents within the tumor tissues and a lower systemic concentration, combined with obstruction of the vessels supplying the tumor. Unlike lipiodol, DEB has the advantages of controlling the level of occlusion and release of the antitumor drug, which can provide a prolonged and sustained drug delivery and a high diffusion of DOX into the liver tissue surrounding the beads.

As the first novel DEB product made in China, CB is a type of ion-exchange bead with some negatively charged functional groups, which has the capability of loading chemotherapeutic agents with a positive charge, such as DOX [18]. A recent report has demonstrated the advantages of CB by comparing the pharmacokinetics and drug release in rabbit liver tissues after TACE with doxorubicin using diverse lipiodol emulsions and CB in rabbit livers [19]. DEB-TACE with CBDOX resulted in a relatively lower concentration of DOX in serum and provided longer release of DOX in tumor tissues from the CB compared to cTACE [19].

The recent introduction of CB in China has provided a valuable choice for patients with unresectable HCC. At present, there have been very few studies comparing DEB-TACE with CB and cTACE in the treatment of Chinese patients with HCC. Our results revealed that patients in the DEB-TACE group tended to present a better response than those in the cTACE group during 6 months of follow-up. OR and DC rates in the DEB-TACE group were all significantly higher than those in the cTACE group at 3 and 6 months. At the same time, this study showed that the OR and DC rates decreased to a certain extent at 6 months compared to those at 3 months in the two groups, which might be related to the formation of collateral circulation and recanalization of tumor vessels. At 1 month after treatment, AFP levels in the DEB-TACE group were significantly lower than those in c-TACE group (*p* = 0.008), indicating a relatively better response to CBs. At the same time, the overall tumor recurrence rate in the DEB-TACE group was significantly lower compared to the cTACE group at 6 months (*p* = 0.036). One potential mechanism to explain our outcomes is that DEB-TACE might contribute to obvious necrosis of the tumors through permanent, super-selective embolization of tumor-feeding arteries, and sustained DOX delivery in tumor tissue. In addition, complete embolization of tumor peripheral blood vessels induced by CB could reduce the probability of recanalization of tumor vessels and formation of collateral circulation, thereby reducing the risk of tumor recurrence and progression.

Several studies have suggested better response using different DEB products compared to cTACE, which is consistent with our research [20, 21]. Malagari et al. [20] used HepaSphere microspheres with a size of 30–60 μm as carriers loaded with DOX to treat HCC. The results showed that the CR and DC rates at 3 months after treatment were 68.9 and 88.9%, respectively. In addition, a previous randomized study conducted by Lammer et al [21] showed a relatively higher rate of CR, OR, and DC in patients who received DEB-TACE with DC Beads compared to cTACE (27 vs. 22%; 52 vs. 44%; and 63 vs. 52%, respectively), with a significant increase in OR rate (*p* = 0.038).

Previous studies have shown that some palliative treatment could downstage HCC, thereby allowing some unresectable HCC to become resectable [22, 23]. In this study, DEB-TACE tended to downstage HCC and create an opportunity for further salvage surgery compared to cTACE (16.7 vs. 6.7%), indicating a relatively higher rate of tumor necrosis and volume reduction. However, there was no statistical significance. It may be related to the small sample size and short follow-up time; some cases may have a chance of salvage surgery with an increased period of treatment.

The most common complication after cTACE is post-embolization syndrome, including fever, abdominal pain, nausea, and vomiting, with an incidence rate from 60 to 80% [24]. In the present study, the most common complication was post-embolization syndrome in both groups, which occurred in 14 patients and 26 patients in the DEB-TACE group and cTACE group, respectively. Despite the higher degree of embolic effect caused by CB, incidence of post-embolization syndrome in the DEB-TACE group was significantly lower compared to the cTACE group (*p* = 0.039), indicating that DEB-TACE with CB is better tolerated in the treatment of HCC. A phase II study demonstrated that DEB-TACE with DOX-loaded DC Beads resulted in decreased toxicity compared to TACE [25]. An elevation of liver enzymes was observed in 31 of 54 patients of the two groups at 1 week after treatment in this study. However, liver toxicity returned to baseline level at 1 month, and parameters of ALT, AST, and TBiL in the DEB-TACE group were better than those in the cTACE group (*p* = 0.045, *p* = 0.040, and *p* = 0.038, respectively).

Previously, several clinical studies in HCC had demonstrated a lower toxicity with DEB-TACE treatment compared to cTACE [21, 26]. The PRECISION V study compared the incidence of complications between DEB-TACE and cTACE, and the results showed that the overall adverse events rate in the DEB-TACE group was significantly lower than that in the cTACE group (20.4 vs. 30.6%). DC Beads showed a better tolerability with a significant reduction in serious liver toxicity (*p* < 0.001) and a significantly lower rate of DOX-related side effects [21]. In our study, the dosage of DOX loaded in the DEB-TACE group was higher than that in cTACE group. However, complications associated with DOX, including myelosuppression and granulocyte reduction, were significantly lower than those in the cTACE group (*p* = 0.015 and *p* = 0.004, respectively), which further demonstrated the sustained and slow release characteristics of CBDOX. The results of this study indicate that DEB-TACE with CBDOX has a relatively higher safety and lower toxicity in the treatment of HCC compared to cTACE.

The current study has several limitations. First, this single-center, retrospective study had a small sample size and was thus underpowered. Conclusions regarding the efficacy of DEB-TACE vs. cTACE cannot be drawn. Second, the downstaging role of the two procedures cannot be accurately compared due to the small sample size and short follow-up time. Undoubtedly, further and larger cohort studies are essential to confirm these preliminary findings. Second, despite the improved response rates demonstrated in the DEB-TACE group, a direct survival benefit cannot be inferred due to the short time of follow-up.

## Conclusion

This study demonstrated that, as the first novel DEB product in China, CB could induce better response and lower tumor recurrence rate in the treatment of Chinese patients with HCC. Furthermore, this regimen was better tolerated and had a relatively higher degree of safety. Undoubtedly, additional large, randomized controlled studies are needed to evaluate this promising therapeutic approach and confirm its long-term efficacy and survival benefit.

## Abbreviations

AFP: Alpha-fetoprotein
ALB: Serum albumin
ALT: Alanine aminotransferase
AST: Aspartate aminotransferase
BCLC: Barcelona clinic liver cancer
CB: CalliSpheres Beads
CR: Complete remission
CT: Computed tomography
DC: Disease control
DEB: Drug-eluting beads
DEB-TACE: Drug-eluting beads transarterial chemoembolization
DOX: Doxorubicin
ECOG PS: Eastern cooperative oncology group performance status
HBV: Hepatitis B virus
HCC: Hepatocellular carcinoma
HCV: Hepatitis C virus
HR: Hepatic resection
mRECIST: Modified response evaluation criteria in solid tumors
MRI: Magnetic resonance imaging
OR: Objective response
PD: Progressive disease
PR: Partial remission
SD: Stable disease
TACE: Transarterial chemoembolization
TBiL: Total bilirubin
TP: Total protein
